# A case report of *Mycobacterium fortuitum* infection after muscle injection

**DOI:** 10.1097/MD.0000000000036060

**Published:** 2023-12-01

**Authors:** Hao Li, Tao Zhang

**Affiliations:** a Clinical laboratory medicine in Pingdingshan Medical District, 989 Hospital of PLA Joint Logistic Support Force, Pingdingshan, Henan, China.

**Keywords:** abscess, case report, infection, *Mycobacterium fortuitum*

## Abstract

**Rationale:**

Injection-related abscesses are a common complication in clinical practice, but the identification of infected bacteria might be difficult.

**Patient concerns:**

A 51-year-old female patient was admitted to the hospital due to a lump on her right buttock that emerged after receiving intramuscular injections to treat left shoulder joint pain. The lump gradually enlarged into a 3.0 to 4.5 cm mass at the time of admission with symptoms such as skin redness, itching, and pain.

**Diagnoses:**

The patient received ultrasonic and other laboratory examinations. Laboratory results from the drainage indicated that the infection was caused by a rapidly growing mycobacteria and was confirmed as *Mycobacterium fortuitum* by matrix-assisted laser desorption/ionization time-of-flight (MALDI-TOF) mass spectrometry.

**Interventions:**

The patient was treated with antibiotics for 12 days after incision and drainage of the abscess in the right buttock. Local dressings were changed regularly. A migration lesion that appeared 3 days after treatment was drained and cleaned when it matured.

**Outcomes:**

The lesion substantially decreased in size and the patient was discharged after 2 months of treatment.

**Lessons:**

Rapidly growing mycobacteria are rare but important pathogens that should be considered in patients with injection-related abscesses. Early identification and appropriate treatment can result in a favorable prognosis.

## 1. Introduction

*Mycobacterium fortuitum* is widely present in the natural environment and is classified as group IV of nontuberculous mycobacteria (NTM). NTMs are opportunistic pathogen that can cause various infections, including skin and soft tissue infections, pulmonary infections, and disseminated infections in immunocompromised individuals.^[1–5]^ Skin and soft tissue infections caused by NTMs often occur after surgery, trauma, or injection.^[6]^ As a subgroup of NTMs, *M fortuitum* is characterized by its weak pathogenicity, non-toxin production, high tolerance and rapid growth compared to other NTM members.^[1]^ Thus, the infections caused by *M fortuitum* can be difficult to diagnose, as the early symptoms are not obvious, and the detection rate of routine culture is low. In primary hospitals, they are often classified as NTMs and cannot carry out intergeneric identification or drug sensitivity tests due to the lack of molecular biology, gene chips and other detection methods, which makes the subsequent treatment difficult. The use of mass spectrometer makes the identification of NTM simple and rapid. Here we report a case of rapid identification of *M fortuitum* using a mass spectrometer.

## 2. Case presentation

A 51-year-old female patient was admitted to the hospital due to a lump on her right buttock that had been present for 1 month. The lump emerged after she had received 4 intramuscular injections including vitamin B12, tanshinones, vitamin K, and other drugs at a private clinic to treat left shoulder joint pain. The injections were administered into the right buttock muscles over a period of 15 days. Following the injections, a spherical hard lump with clear borders appeared at the injection site, without redness or obvious tenderness. The lumpgradually became soft and enlarged into a 3.0 to 4.5 cm mass 35 days after the first discovery, along with symptoms such as skin redness, itching, and pan. The patient self-administered various medications, including spiramycin tablets, Sanjie tablets, amoxicillin capsules, and Xiaoyao pills, to treat the symptoms, but with no relief, and in contrast the area of redness and local pain increased.

An ultrasonic examination revealed a thickened subcutaneous fat area in the right upper outer buttocks, with enhanced echo and an irregular liquid dark areameasuring approximately 6.3 × 3.2 × 3.3 cm. Granular substance could be seen when the area was pressed. No obvious abnormalities were found in peripheral blood or other routine tests, including coagulation or liver or kidney functions.

**Figure 1.**
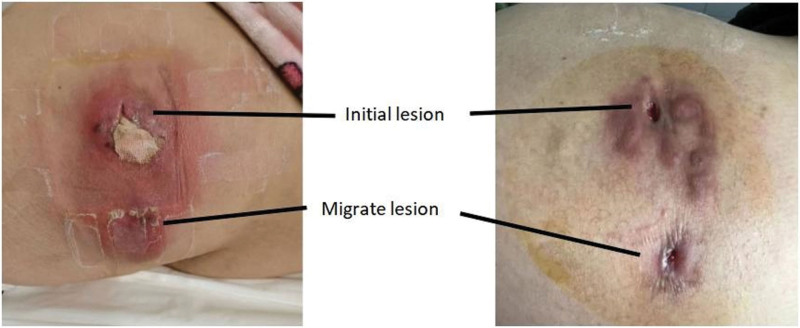
*Left*, initial (top) and new metastatic (bottom) lesions after incision drainage of the primary lesion. *Right*, two month after treatment.

One day following admission, the patient underwent an incision to drain the abscess in the right buttock under local anesthesia, resulting in a drainage of about 70 mL of thick yellow pus without noticeable peculiar odor. The abscess cavity was rinsed with hydrogen peroxide and normal saline, after which a drainage tube was placed to drain the pus, and a sterile cotton pad was used to cover the wound. Local dressings were changed daily. The patient received intravenous administration of piperacillin/tazobactam (4.5 g per 8 hours) and ornidazole (0.5g per 12 hours). After 3 days of treatment, a new induration was formed 5 cm directly below the original abscess site, which was considered as a migration lesion from the initial site (Fig. 1). After the new lesion matured, the necrotic tissue was removed, and the sinus was cleaned. The anti-infection treatment was continued for 12 days, and the local dressings were changed every 2 days a month later.

**Figure 2.**
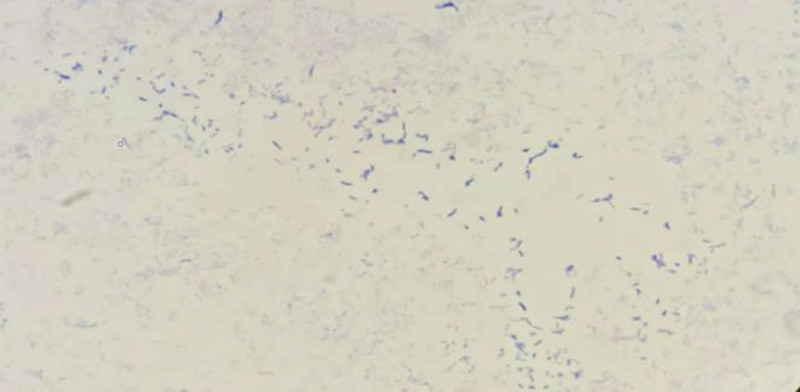
Weakly Gram stained bacilli detected from the pus sample.

**Figure 3.**
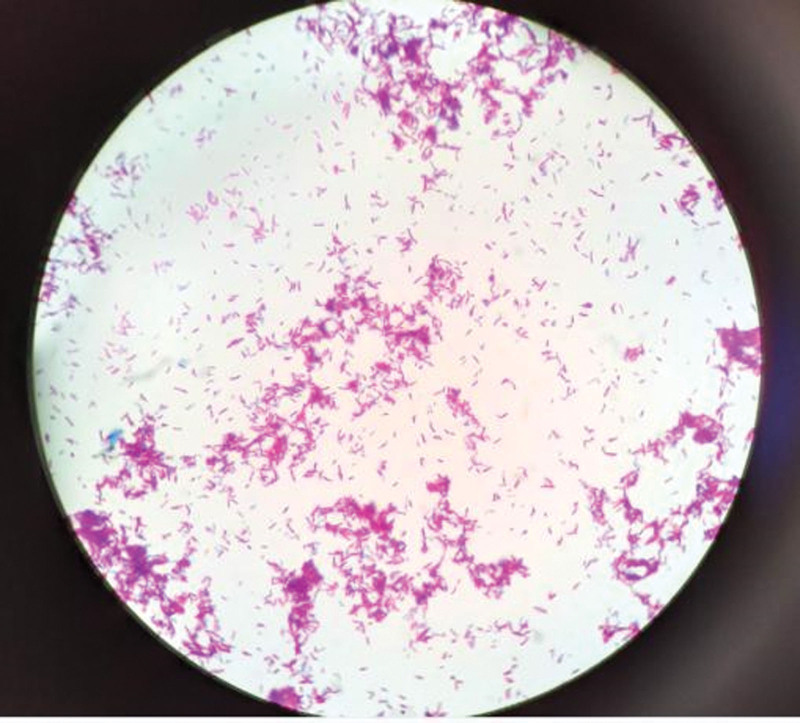
Acid-fast staining shows rod-shaped red bacteria.

The pus sample was inoculated onto blood agar, MacConkey agar, and chocolate agar plates respectively and incubated at 35°C. Additionally, 2 slides were prepared for staining with the pus sample. After 72 hours, small colonies were observed on the blood agar and chocolate agar plates. These colonies appeared as short bacilli with weak Gram staining (Fig. 2) and acid-fast positive when stained with the Ziehl–Neelsen (Fig. 3). A small number of acid-fast positive short rods were also observed on the direct-smeared slides.

**Figure 4.**
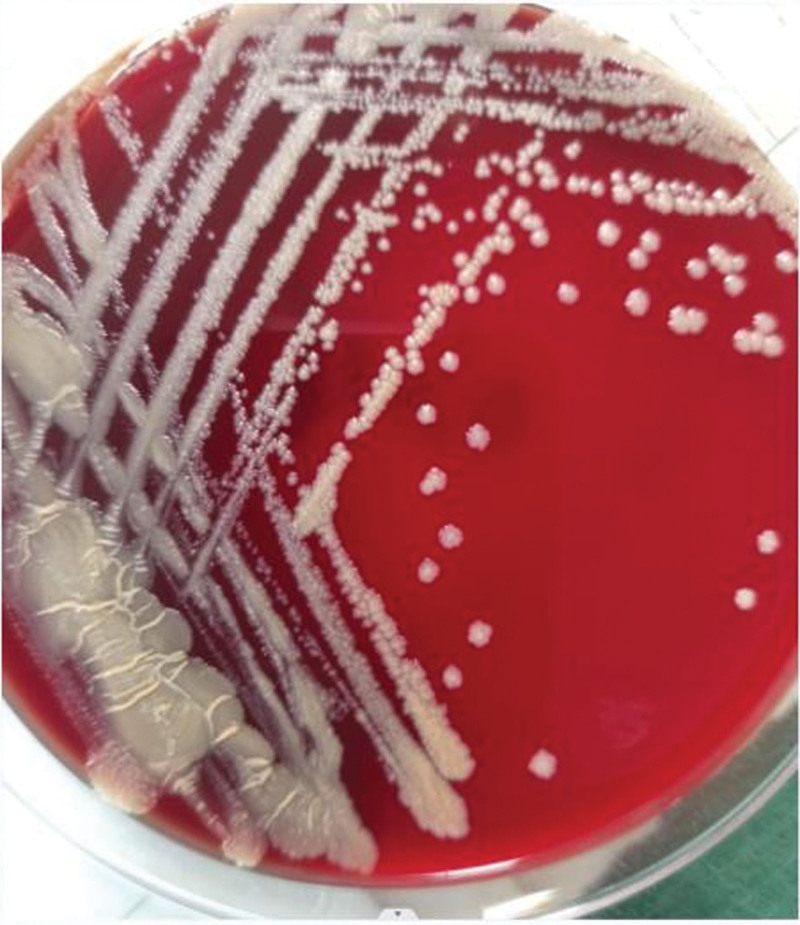
Colony morphology after 7 days of cultivation.

The colonies grew gradually over the following 5 days to a diameter of 0.5 to 1 mm with a white, moist, and smooth appearance. By day 7, the white colonies had increased to approximately 2 to 3 mm in diameter and exhibited a round, smooth, and petal-shaped morphology (Fig. 4). No bacterial growth was discovered in ordinary and anaerobic cultures. The bacteria showed positive results in the MacConkey agar culture, arylsulfatase test, 68°C catalase test, urease test, nitrate reduction test, and NaCl tolerance test. Negative results were obtained for the utilization of mannitol, sorbitol, inositol, and citrate salts.

**Figure 5.**
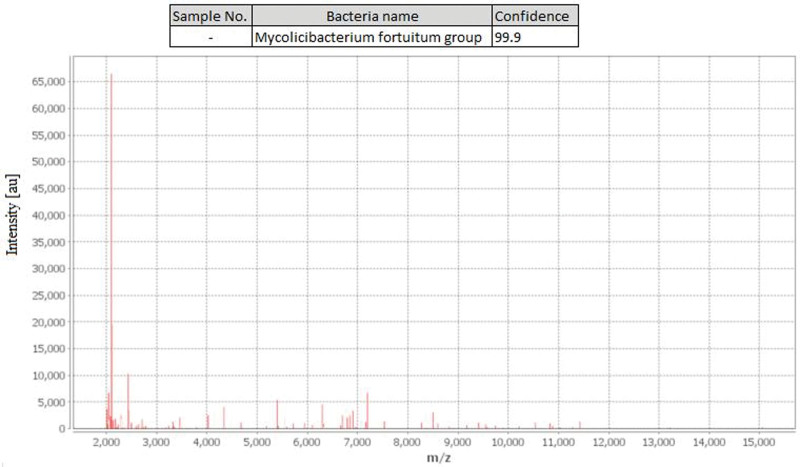
The colony was identified as *Mycobacterium fortuitum* by mass spectrometry. Top, the output of the MS; bottom, raw spectral fingerprints obtained from the colony.

Thus, based on the growth rate, pigment production, enzyme activity, and growth on different media, the bacteria were consistent with the characteristics of rapidly growing mycobacteria, which are the majority of NTM infections reported in China.^[7–9]^ They were further identified as *M fortuitum* (> 99.9%, Fig. 5) using matrix-assisted laser desorption/ionization time-of-flight mass spectrometry (Biomerieux, France). Minimal inhibitory concentration testing revealed that the bacteria were sensitive to amikacin, gentamicin, ciprofloxacin, levofloxacin, and clarithromycin but resistant to erythromycin, penicillin, ampicillin, cefotaxime, vancomycin, and co-trimoxazole.

After reviewing the laboratory results, the treatment plan was deemed effective and continued. Following 2 months of continuous treatment, the lesion substantially decreased in size, as shown in Figure 2 (right). As a result, the patient was discharged from the hospital in a stable condition and follow-up appointments were scheduled to monitor the healing progress.

## 3. Discussion

The clinical signs of *M fortuitum* infection varies widely, ranging from localized abscesses to systemic infections. In our patient’s case, the infection presented as a rapidly enlarging abscess in the right buttock, which was initially treated with incision and drainage. However, a new lesion appeared after 3 days oftreatment, which was considered a migration lesion from the initial site. This phenomenon is not uncommon in NTM infections, and it highlights the importance of a thorough evaluation of the patient for successful treatment.

Diagnosis of *M fortuitum* infection can be challenging, as it is a fastidious organism that requires special culture techniques and prolonged incubation periods for growth. Acid-fast staining and molecular methods such as polymerase chain reaction can aid in the diagnosis of *M fortuitum* infection. In our patient’s case, the diagnosis was confirmed by culturing the pus on specific media and subsequent identification by matrix-assisted laser desorption/ionization time-of-flight mass spectrometry.

Treatment of NTM infection is challenging, as it is often resistant to multiple antibiotics, has a long duration, and is prone to relapse.^[10]^ Antimicrobial susceptibility testing is essential to guide appropriate treatment, and a combination of antibiotics is often necessary to achieve a successful outcome.^[11,12]^ In our patient’s case, the organism was sensitive to amikacin, gentamicin, ciprofloxacin, levofloxacin, and clarithromycin, and the patient was treated with a combination of piperacillin/tazobactam and ornidazole, which effectively improved the patient’s symptoms.

It is noteworthy that the resistance of *M fortuitum* strains varies geographically, and the choice of antibiotics should be based on local antimicrobial susceptibility data. A study by Zheng et al^[13]^ has shown that in China, the resistance of *M fortuitum* strain is lower to moxifloxacin and amikacin, but significantly higher to macrolides, compared to that reported in the studies by Shahraki et al, and Gleeson et al^[5,14]^ This might be related to the frequent use of macrolides by Chinese doctors to treat respiratory mycoplasma infections, leading to increased exposure to the drug.^[15,16]^

## 4. Conclusion

*M fortuitum* infection should be considered in patients presenting with skin and soft tissue infections, especially after injections, surgery, or trauma. A thorough evaluation, including appropriate diagnostic testing and antimicrobial susceptibility testing, is essential to guide appropriate treatment and prevent recurrence. The choice of antibiotics should be based on local susceptibility data, and a combination of antibiotics may be necessary for successful treatment.

## Author contributions

**Data curation:** Tao Zhang, Hao Li.

**Formal analysis:** Tao Zhang, Hao Li.

**Investigation:** Tao Zhang, Hao Li.

**Methodology:** Tao Zhang, Hao Li.

**Project administration:** Hao Li.

**Resources:** Hao Li.

**Supervision:** Hao Li.

**Validation:** Tao Zhang.

**Writing – original draft:** Tao Zhang.

**Writing – review & editing:** Hao Li, Tao Zhang.
